# Antidepressants in association with reducing risk of oral cancer occurrence: a nationwide population-based cohort and nested case-control studies

**DOI:** 10.18632/oncotarget.7049

**Published:** 2016-01-28

**Authors:** Chia-Min Chung, Tzer-Min Kuo, Shang-Lun Chiang, Zhi-Hong Wang, Chung-Chieh Hung, Hsien-Yuan Lane, Chiu-Shong Liu, Ying-Chin Ko

**Affiliations:** ^1^ Environment-Omics-Disease Research Center, China Medical University Hospital, Taichung, Taiwan; ^2^ Graduate Institute of Clinical Medical Science, China Medical University, Taichung, Taiwan; ^3^ Department of Health Risk Management, College of Public Health, China Medical University, Taichung, Taiwan; ^4^ Department of Psychiatry, China Medical University Hospital, Taichung, Taiwan; ^5^ Department of Family Medicine, China Medical University Hospital, Taichung, Taiwan

**Keywords:** oral cancer, antidepressants, cohort

## Abstract

**Objectives:**

Several studies suggested that antidepressant use may increase or decrease the risk of cancer occurrence, depending on specific cancer types. The possible carcinogenic effect of antidepressants has received substantial attention; however, evidence remains inconclusive. Here we investigated associations between the use of antidepressants and occurrences of oral cancer (OC).

**Methods:**

Two million samples were randomly collected from the National Health Insurance Research Database in Taiwan, which covers 98% of the total population (23 million). All patients from2000 to 2009 were followed up. We identified 5103 patients newly diagnosed with OC after antidepressants use in addition to 20,412 non-OC matched subjects and 95,452 unmatched non-OC subjects.

**Results:**

In nested case control analysis, factors associating with OC, including age [OR = 1.02; 95% confidence interval (CI) = 1.01–1.03) and male (OR = 5.30; 95% CI = 4.92–5.70) were independently associated with increased risk of OC. Based on the functions of antidepressants, antidepressants treatment medications were further classified to investigate risk of OC. Selective serotonin reuptake inhibitors (OR = 0.61; 95% CI = 0.53–0.70) and tricyclic antidepressants (OR = 0.57; 95% CI = 0.52–0.63) were associated with reduced risk of OC. The risk of developing OC among subjects taking antidepressants was less than 26% [hazard ratio (HR) =0.74; 95% CI = 0.68–0.81] in prospective cohort study. The effect of a cumulative duration and dose was a significantly reduced risk of OC.

**Conclusions:**

The association between antidepressant use and decreasing OC risk were demonstrated by both prospective and nested case–control studies.

## INTRODUCTION

Antidepressants are used widely in the general population [1, 2] and are frequently used among cancer patients for the treatment of various disorders such as depression, anxiety, insomnia, and chronic pain (including cancer pain) [3, 4]. The use of antidepressants has been steadily increasing not only in the elderly but also in young and middle-aged populations [5]. In Western countries, more than 10% of the US population are receiving at least one prescription every year [6], and antidepressants were among the top five prescription medications used between ages 45 and 64 (17% among women and 8% among men) from 2007 to 2011 [7] in Canada.

Since the early 1990s, several studies both in tumor cell cultures and animal models have hypothesized a potential association between antidepressant use and cancer. It was first proposed that antidepressants could increase the risk of cancer, with breast and colon cancers being the most studied malignancies. In breast cancer, an increased risk was suggested after the long-term use of tricyclic antidepressants (TCAs) and selective serotonin reuptake inhibitors (SSRIs) [8–11]; however, there was no change in the risk in other reports [12–17]. For colon cancer, some studies demonstrated a decreased risk with SSRI exposure or with overall antidepressant therapy, [18–20] whereas others showed either no difference in risk [21, 22] or even increased risk [23]. Other epidemiological evidences support an increased risk of all cancers including lung, ovarian, and prostate cancers, with the use of antidepressants [10, 24, 25]; however, some studies showed no difference in risk [23, 26]. The possible carcinogenic effect of antidepressants has received substantial attention; however, evidence in this regard remains inconclusive.

To our knowledge, no large population-based study has evaluated oral cancer (OC) risk and antidepressant use thus far. OC in Taiwan has been reported to have the highest rate of incidence worldwide in 2008 and 2012 by the International Agency for Research on Cancer series [27, 28]. Because of the high prevalence of antidepressant use and OC and their possible biological activity in cancer-related pathways, a unique setting is created to study the association between antidepressant and OC. In this study, we investigated the possible associations between the use of antidepressants and the occurrence of OC in a nationwide population-basedcohort and in nested case–control studies.

## RESULTS

**Figure 1:**
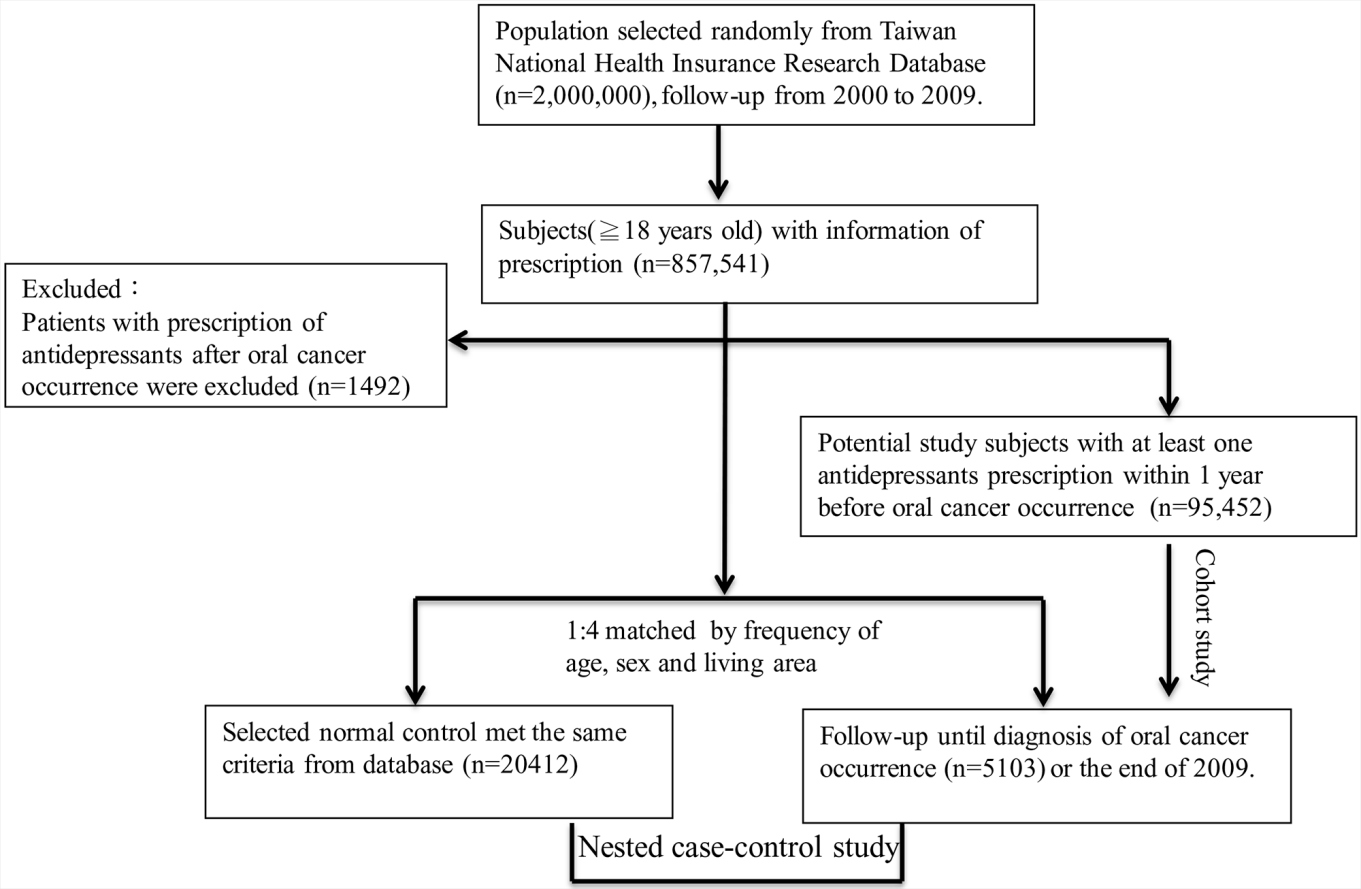
Schematic of the samples selection process for the antidepressants prescription and oral cancer occurrence

Data of study subjects were obtained from among patients with recorded OC and antidepressant prescriptions in Taiwan NHIRD (Figure 1). A total of 5,103 newly diagnosed OC patients (mean age 56.6 ± 13.9 years, male 83.6%) were identified from the 2,000,000 sampling cohort data set between January 2000 and December 2009. Another 20,412 non-OC subjects matched with age, sex, and living area, were enrolled as non-exposure controls. The demographic parameters of study subjects are shown in Table 1. Age, sex, geographical area, and urbanization status were not significantly different between patients with and without OC. Patients with OC were less likely than controls to have ever used any antidepressants, monoamine oxidase inhibitors (MAOIs; 0.12% vs. 0.23%, *p* = 0.1333), SSRIs (4.86% vs. 7.76%, *p* < 0.001), or TCAs (6.43% vs. 10.51%, *p* < 0.001) (Table 1).

**Table 1:** Demographic data of the patients with and without oral cancer in the nested case control study

Variables	Oral cancer		*p*-value
	Yes(n=5103)	No(n=20412)	
Age, years(Mean±SD)	56.6±13.9	56.6±14.0	NS
Age group(years)			
18≦age<65, n(%)	3646(71.45)	14584(71.45)	
65≦age<75, n(%)	852(16.70)	3408(16.70)	
75≦age, n(%)	605(11.86)	2420(11.86)	
Male, n(%)	4267(83.62)	17068(83.62)	NS
Geographic area, n(%)			
North	2410(47.22)	9640(47.22)	NS
Central	301(5.90)	1204(5.90)	NS
South	2236(43.82)	8944(43.82)	NS
East	156(3.06)	624(3.06)	
Urbanization status, n(%)			
Metropolis	3215(63.00)	12205(63.00)	NS
Satellite city/town	1717(33.65)	7499(33.65)	NS
Rural area	171(3.35)	708(3.35)	NS
Antidepressants, n (%)	625(12.25)	4206(20.61)	<.0001
Others	98(1.95)	616(3.02)	0.0003
MAOIs	6(0.12)	47(0.23)	0.113
SSRIs	248(4.86)	1581(7.76)	<.0001
TCAs	454(8.89)	2827(14.09)	<.0001

Abbreviations: NS, not significant; SD, standard deviation; MAOIs, Monoamine oxidase inhibitors; SSRI, selective Serotonin Reuptake Inhibitors; TCAs, Tricyclic antidepressants. Others include Serotonin Norepinephrine Reuptake Inhibitors, Mirtazapine and Norepinephrine and dopamine reuptake inhibitors.

To investigate the independent factors associated with the risk of developing OC, a logistic regression analysis was conducted; age (OR 1.02; 95% CI, 1.01–1.03, *p* < 0.0001), male (OR, 5.30; 95% CI, 4.92–5.70, *p* < 0.0001), geographic area (Table 2) and alcoholism(OR, 2.01; 95% CI, 1.53–2.65, *p* < 0.0001), tobacco use disorder (OR, 4.99; 95% CI, 1.34–18.61, *p* < =0.0017); Supplementary Table S2) were independently associated with increased risk of OC. Subjects with antidepressant medication had a reduced risk of OC (OR, 0.53; 95% CI, 0.48–0.57, *p* < 0.0001; Table 2). Based on their mechanism of action, antidepressants were further classified to investigate the risk of OC. SSRIs (OR, 0.61; 95% CI, 0.53–0.70, *p* < 0.0001) and TCAs (OR, 0.57; 95% CI, 0.52–0.63, *p* < 0.0001) were associated with a decreased risk of OC. After matching for age, sex, geographical area, and urbanization status, these antidepressants were still associated with decreased risk of OC. No statistically significant association between current MAOI therapy and OC risk (OR, 0.51; 95% CI, 0.22–1.19; Table 2) was detected.

**Table 2:** Antidepressants use associated with oral cancer occurrence by nested case-control study(OR)* and cohort study (HR)^+^

Variables	OR(95% CI)	HR (95% CI)^+^
Age, years	1.02(1.01-1.03)^#^	-----
Male	5.30(4.92-5.70)^#^	-----
Urbanization	1.25(1.09-1.44)^#^	-----
Rural area	0.94(0.81-1.09)^#^	-----
Anti-depressants	0.53(0.48-0.57)*	0.74(0.68-0.81)
MAOIs	0.51(0.22-1.19)*	0.59(0.24-1.41)
SSRIs	0.61(0.53-0.70)*	0.76 (0.68-0.90)
TCAs	0.57(0.52-0.63)*	0.79(0.68-0.84)

Abbreviations: R= odds ratio, HR = hazard ratio, CI = confidence interval.

# The logistic regression analysis was conducted to investigate the independent factors associated with the risk of OC occurrence

* The conditional logistic model was used to examine association between antidepressants use and oral cancer occurrence.

+ The Cox proportional hazard model was used to examine risk between antidepressants use and oral cancer occurrence prospectively use after adjusting for age, gender and living area by cohort study.

Subjects were divided into two groups according to whether they took antidepressants to assess the independent effects of antidepressants on the risk of OC in the prospective study. The Cox proportional hazard regression analysis revealed that the risk of developing OC among subjects taking antidepressants was less than 26%, compared with subjects who were not on antidepressants [hazard ratio (HR), 0.74; 95% CI, 0.68–0.81] after adjustment for age, sex, and geographical area. The risk of OC was similar among subjects who exclusively used SSRIs (HR, 0.74; 95% CI, 0.68–0.81) or TCAs (HR, 0.79; 95% CI, 0.68–0.84; Table 2).

Among users of SSRI and TCA with treatment initiation more than 1 year and less than 3 years before the index date, there was a lower risk of OC, with ORs of 0.86 (95% CI, 0.73–1.01) and 0.87 (95% CI, 0.74–1.03), respectively. However, among MAOI users with treatment initiation more than 1 year and less than 3 years before the index date, there was no significant association with the risk of OC(OR, 0.98; 95% CI, 0.82–1.50). Subjects with SSRI and TCA treatment more than 5 years had a lower risk of OC (OR=0.21; 95% CI, 0.16-0.27 and OR=0.29; 95% CI=0.23-0.36 respectively). (Table 3). Compared with non antidepressant user, SSRIs (low dose OR =0.70; 95% CI, 0.57–0.85; high dose OR =0.55; 95% CI, 0.46–0.66) and TCA (low dose OR =0.71; 95% CI, 0.61–0.83; high dose OR =0.53; 95% CI, 0.46–0.61) was associated with decreased risk of OC occurrence in a dose- response manner. However, treatment with MAOIs was not associated with the risk of OC occurrence (Table 4). We also found that subjects with SSIR or TCA medication had lower risk for alcoholism compared with other antidepressants group(OR =0.61; 95% CI, 0.53-0.70 and OR =0.12; 95% CI, 0.08–0.19 respectively). Due to small sample sizes, the association between tobacco use disorder and antidepressant was not significantly (Supplementary Table S3).

**Table 3:** Multivariable analysis among users of antidepressants, looking at different levels of cumulative duration

Antidepressants type	Timing of first prescription before index date	OR (95% CI)
TCAs	unexposed	1
TCAs	1=< years <3	0.87(0.74-1.03)
TCAs	3=< years <5	0.78(0.67-0.92)*
TCAs	years >=5	0.29(0.23-0.36)*
SSRIs	unexposed	1
SSRIs	1=< years <3	0.86(0.73-1.01)
SSRIs	3=< years <5	0.75(0.65-0.87) *
SSRIs	years >=5	0.21(0.16-0.27) *
MAOIs	unexposed	1
MAOIs	1=< years <3	0.98(0.82-1.50)
MAOIs	3=< years <5	0.84(0.71-1.27)
MAOIs	years >=5	0.28(0.03-2.55)

The logistic model was used to examine association between antidepressants use and oral cancer occurrence after adjusting for age, gender and living area by nested case-control study.

* P<0.05

**Table 4:** Risk for oral cancer occurrence associated with dose response of different classes of antidepressant

	Case	Control	
Antidepressant Use	N (%)	N (%)	OR(95% CI)
Nonuse of antidepressant	4650(91.12)	17511(85.91)	1
SSRIs			
≤0.5DDD(Low dose)	115(2.25)	641(3.14)	0.70(0.57-0.85)
>0.5DDD(High dose)	133(2.61)	940(4.61)	0.55(0.46-0.66)
TCAs			
≤0.5DDD(Low dose)	197(3.86)	1049(5.15)	0.71(0.61-0.83)
>0.5DDD(High dose)	256(5.02)	1823(8.94)	0.53(0.46-0.61)
MAOIs			
≤0.5DDD(Low dose)	6(0.1)	47(0.23)	0.57(0.2-1.63)

Abbreviations: DDD=defined daily dose; SSRI, selective Serotonin Reuptake Inhibitors; TCAs, Tricyclic antidepressants; MAOIs, Monoamine oxidase inhibitors;

## DISCUSSION

Our current study revealed that subjects taking antidepressants had an associated decrease in the risk of OC occurrence in both prospective and nested case–control studies after a maximum 10-year follow-up.

To our knowledge, this is the first epidemiological study to report an association between the use of antidepressant and risk of OC. Serotonin was thought previously to have a role in carcinogenesis; SSRIs would thus inhibit cancer initiation [29]. Studies have shown that SSRIs are capable of inducing apoptosis in tumor cell [30]. Additionally, approximately 90% of oral cancers are squamous cell carcinoma. Kinjo et al. also demonstrated SSRIs have antiproliferative effect in squamous carcinoma [31]. We believe that SSRIs could retard the growth of developed tumors by inhibiting tumor cell proliferation [32]. We observed a decreased cancer risk in subjects with a more than 3-year duration of SSRI use to support that SSRIs have an anti-enhancer effect rather than an anti-initiation effect on OC. Taken together, these results suggest that antidepressants primarily exert an inhibitory effect on tumor growth, and this is consistent with findings from the cell and animal models.

We hypothesized that the potential mechanism of action of antidepressants in decreasing the risk of OC is via a reduction of the usage of betel quid (BQ) or cigarettes. Some studies have reported that therapy with nortriptyline, a TCA, appears to be effective in the treatment of nicotine addiction [33, 34]. Spring et al. found that fluoxetine, a SSRI, initially increased smoking cessation among smokers with a history of depressive disorders, [35] and Brown et al. found it significantly impaired smoking cessation in a group receiving 10 weeks of treatment with a combination of fluoxetine and nicotine replacement therapy in smokers with elevated depressive symptoms [36]; however, other studies have not detect a significant effect [37, 38]. There was no evidence of a significant effect of MAOIs on smoking cessation [39]. The potential mechanism is related to the effect of nicotine on increasing the release of serotonin in the brain, and there is strong evidence that serotonergic tone plays a role in the effects of nicotine [40]. Nortriptyline and SSRIs have been shown to inhibit serotonin reuptake by blocking the serotonin transporter, thus probably decreasing the risk of smoking initiation and nicotine dependence and increasing success of smoking cessation. The other mechanisms for smoking cessation include dopaminergic, noradrenergic, or nicotinic–cholinergic monoaminergic activity or blocking of nicotine receptors. However, the most important mechanism for smoking cessation efficacy is unclear. If noradrenergic effects are important in treatments for smoking cessation, then MAOIs and other TCAs should be effective; however, the results of clinical trials are controversial due to the few trials and small sample sizes [34].

BQ chewing was reported to be an independent risk factor, with an attributable risk accounting for 66%–79% of patients with OC [41, 42]. The attributable fraction, combined from alcohol, BQ, and cigarette use, to cancers of the pharynx and larynx were 93% [43]. The antidepressant properties of BQ and arecoline have been reported to directly inhibit MAO-A enzymatic activity in cell, rat, and human models [44–47]. MAO-A metabolizes various primary, secondary, and tertiary monoamines and preferentially deaminates neurotransmitters relative to depression. MAOIs are used in the treatment of clinical depression and may be used for BQ cessation. The other antidepressants were not reported to have efficacy in for BQ cessation.

The use of MAOIs for the treatment of depression in clinical practice is low in our database because of their known adverse effects from drug and food interactions and the resulting need for dietary restrictions and careful medical management. Because the sample size of MAOI users is small, our data do not support the hypothesis that MAOI antidepressants decreased the risk of oral cancer in the nested case–control studies. However, the trends of decreased risk of OC are similar to that with other antidepressants.

This study had several limitations. First, possible causes of OC, for which information are unavailable for retrieval from the database were not adjusted for. Unhealthy lifestyles are known risk factor for OC and some of these lifestyle factors—for example, BQ chewing, cigarette smoking, and current status of alcohol consumption—might be associated with depression. Therefore, antidepressant use might be associated with these risk factors. Although we had adjustments for alcoholism and tobacco use disorder in the statistical model, we cannot reduce confounding effects caused by BQ chewing, cigarette smoking, and current status of alcohol consumption. Second, subjects with antidepressant prescription records may not actually take the medications, potentially causing an underestimated risk for antidepressant. Third, adherence to antidepressants therapy was and treatment duration was short in Taiwan. Mean use of time duration of SSRI and TCA were 1.81 years and 1.68 years respectively (Supplementary Table S1). We are not able to examine the long term effects of antidepressants on risk of OC.

We assessed the effect of different classes of antidepressant (i.e., TCAs and SSRIs) for which we had clear a priori hypotheses. We did not analyze individual drugs, such as fluoxetine and nortriptyline, within each class. Such analyses would increase the number of statistical tests and thus the risk of detecting spurious associations. Selection and recall biases were unlikely in this study. Information was identified from the Taiwan NHIRD, which includes almost all cases of OC and antidepressant use in the random source population. The loss-to-follow-up rate is low, due to its compulsive characteristics, management by the government, and high coverage of the entire population.

The association between antidepressant use and decreased risk of OC was demonstrated by both prospective and nested case–control studies. We found that subjects with SSIR or TCA medication had lower risk for alcoholism. No information regarding smoking status was available in the NHIRD. The relatively small sample size of tobacco use disorder was not able to detect antidepressant effects on smoking cessation. We hypothesized that antidepressant use may help reduce or terminate alcohol, BQ, and cigarette use and consequently OC occurrence. These results should be confirmed by larger prospective studies and further research. Further investigation of the association between antidepressants and risk of OC is warranted because of the widespread use of SSRIs and TCAs. Clinical investigation of the effect of SSRIs and TCAs in decreasing the risks of cancer recurrence, metastasis, and death could be an interesting topic for future research.

## MATERIALS AND METHODS

### Data sources

Data analyzed in this study were retrieved from the Taiwan National Health Insurance Research Database (NHIRD), which was established in 1995 and collects all claims of those insured under the National Health Insurance (NHI) program. The program covers more than 98% of the total population (23,000,000) and has contracted with 97% of the hospitals and clinics in Taiwan [48]. Recently, the Taiwan NHIRD comprised claims data of 2,000,000 individuals randomly selected from all insured enrollees. This sample represents the original medical claims for all islanders covered under the NHI program. These new well-defined data sets are more powerful compared with previous data sets encompassing 1,000,000 individuals. It has twice their sample size and can be linked to death certificate files managed by the Ministry of Health and Welfare in Taiwan to increase the follow-up rate to more than 90%.

Diagnoses were coded according to the *International Classification of Disease, Ninth Revision, Clinical Modification* (ICD-9-CM). The database used in this study can be interlinked by the scrambled, unique, individual personal identification number. The NHRI safeguards the privacy and confidentiality of all beneficiaries and transfers health insurance data to health researchers after ethical approval has been obtained. In this analysis, access of the NHIRD has been approved by the CMU Ethics Review Committee.

### Study patients

To concentrate our study sample to the adult population, we only selected patients older than 18 years. In this study, we identified OC of the oral cavity and pharynx, including cancers of the lip, tongue, gum, floor of the mouth, and other parts of the oral cavity (ICD-9-CM, 140,141–145) as well as of the oropharynx, hypopharynx, and other parts of the pharynx (ICD-9-CM, 146, 148–149). ICD-9 code 305.1 is coding fortobacco use disorder and ICD-9 code 303.9x and 305.xx is coding for alcoholism. Patients with OC diagnosed prior to 2000 were excluded from this study. Newly diagnosed OC patients were identified from the cohort database since January 1, 2000. Data for a total of 857,541 patients with prescription information were included. To prevent a temporal–causal relation between antidepressants and OC, patients with prescription for antidepressants after OC occurrence were excluded (*n* = 1492). A total of 95,452 study subjects with at least one antidepressant prescription within one year before OC occurrence were retrieved from Taiwan NHIRD after excluding subjects with missing information on age or sex (Figure 1). We used a systematic random sampling method; a method widely used for preventing selection bias in databases with large sample sizes widely used to match four insured people without OC during the same period. To adjust known risk factors across groups, we considered the following variables, including the time when subjects were enrolled: age, sex, and living area. All patients were followed up to December 31, 2009. The endpoint of the study was the occurrence of OC. We identified 5,103 patients newly diagnosed with OC and 20,412 non-OC matched subjects. A nested case–control study was conducted to evaluate the relationship between the use of antidepressants and risk of OC.

The exposure variable in this study was antidepressant medication, which was retrieved from the medical expenditure and prescription claims data in the national database. The presence of exposure to an antidepressant was defined as the prescription of a particular antidepressant for at least 1 month during the case or control time periods. All included individual antidepressants by each class consist of (1) TCAs: imipramine, amitriptyline, clomipramine, dothiepin, and maprotiline; (2) SSRIs: fluoxetine, paroxetine, sertraline. fluvoxamine, citalopram, and escitalopram; (3) MAOIs: moclobemide; (4) other antidepressants: mirtazapine, venlafaxine, trazodone, bupropion, and duloxetine. Nonusers were defined as those without any antidepressant prescription in the year preceding the index date. The more detailed information was shown in the Supplementary Table S1. Based on definition of daily dose (DDD) defined by WHO, the mean daily dose of all antidepressants prescribed within the 90 days of the last prescription was calculated and categorized as low dose (≤ 0.5 DDD) and high dose (> 0.5 DDD) [49].

### Statistical analysis

All data were expressed as the frequency (%) or mean ± standard deviation. Parametric continuous data between the OC and non-OC subjects were compared by unpaired Student's *t*-test. The categorical data between the two groups were compared with chi-square and Fisher's exact tests as appropriate. To estimate the odds ratios (ORs) and their 95% confidence intervals (CIs) in the nested case–control study, a conditional logistic model was used to examine associations between OC occurrence and antidepressant use.

Subjects were classified into two groups according to whether they used antidepressants in this prospective study. To assess the independent effects of antidepressants on the occurrence of OC, we conducted Cox proportional hazard regression models in all of the patients using data on age, sex, and geographical area. Statistical analysis was conducted utilizing SAS9.3 software (Cary, North Carolina, USA).

## SUPPLEMENTARY TABLES


