# Supplementary material for: Antidepressants in association with reducing risk of oral cancer occurrence: a nationwide population-based cohort and nested case-control studies
Source: Oncotarget. 2016 Jan 28;7(10):11687–95. doi: 10.18632/oncotarget.7049 (PMC4905503; doi:10.18632/oncotarget.7049)
Supplement: Supplementary file 1 [file oncotarget-07-11687-s001.pdf]

SUPPLEMENTARY TABLES

Supplementary Table S1: The antidepressants class and prescriptions in subjects with age≧18 years old

Class	Mean prescriptions	Mean use of time duration(Year)	Antidepressants agents	N	%
**Tricyclics**	10.07 ± 19.6	1.68± 2.56			
			Imipramine	30327	31.77
			Amitriptyline	22283	23.34
			Dothiepin	821	0.86
			Maprotiline	110	0.12
**SSRIs**	12.78 ± 17.94	1.81 ± 2.55			
			Fluoxetine	12350	12.94
			Paroxetine	9436	9.89
			Sertraline	8199	8.59
			Fluvoxamine	2270	2.38
			Escitalopram	1531	1.60
**MAOIs**	12.3 ± 19.33	1.08 ± 1.78			
			Moclobemide	271	0.28
**Others**	11.21 ± 18.80	1.47 ± 2.29			
			Mirtazapine	1937	2.03
			Venlafaxine	1787	1.87
			Trazodone	1759	1.84
			Bupropion	1639	1.72
			Duloxetine	732	0.77

TCAs, Tricyclic antidepressants; SSRIs: selective Serotonin Reuptake Inhibitors; MAOIs : Monoamine oxidase inhibitors

Supplementary Table S2: Substance use associated with oral cancer occurrence by nested case-control study (OR)

Variables	Oral cancer		OR(95% CI)
	**Yes(n = 5103)**	**No(n = 20412)**	
Alcoholism n(%)	77(1.51)	154(0.76)	2.01(1.53–2.65)
Tobacco use disorder n(%)	5(0.1)	4(0.02)	4.99(1.34–18.61)

ICD-9 code 303.9x and 305.xx is coding for alcoholism.
ICD-9 code 305.1 is coding for tobacco use disorder.

Supplementary Table S3: Substance use associated with Antidepressants use

Antidepressant Use	OR(95% CI)
Alcoholism	
Other	1
SSRIs	0.61(0.53–0.70)
TCAs	0.12(0.08–0.19)
Tobacco use disorder	
Other	1
SSRIs	2.22(0.2–24.42)
TCAs	0.33(0.04–2.735)

TCAs, Tricyclic antidepressants; SSRIs: selective Serotonin Reuptake Inhibitors;
